# Skin Lesions in Children: Evaluation of Clinicopathological Findings

**DOI:** 10.5146/tjpath.2023.01599

**Published:** 2023-09-15

**Authors:** Begum Calım-Gurbuz, Burcin Pehlıvanoglu, Tuce Soylemez-Akkurt, Ozan Erdem, Anvar Ahmedov

**Affiliations:** Department of Pathology, Basaksehir Cam and Sakura City Hospital, Istanbul, Turkey; Dokuz Eylul University Faculty of Medicine, Izmir, Turkey; Department of Dermatology, Reconstructive and Aesthetic Surgery, Basaksehir Cam and Sakura City Hospital, Istanbul, Turkey; Department of Plastic, Reconstructive and Aesthetic Surgery, Basaksehir Cam and Sakura City Hospital, Istanbul, Turkey

**Keywords:** Children, Skin, Neoplastic, Inflammatory, Lesion

## Abstract

***Objective:*** Pediatric skin diseases may show various manifestations, occasionally affecting the patients’ quality of life. Histopathological examination may be required for the diagnosis. The aim of this study was to evaluate the spectrum of clinicopathological features in pediatric skin lesions.

***Material and Methods:*** A total of 368 biopsies of 359 consecutive patients were included. The clinicopathological findings were retrospectively evaluated. Non-neoplastic (inflammatory) lesions (ILs) (n=186) were grouped per their origin, while neoplastic/proliferative lesions (NPLs) (n=182) were grouped based on their pattern. The clinical and histopathological characteristics were statistically analyzed.

***Results:*** 51% were male and the median age was 10.4±4.9 years (range 0-17). ILs mainly involved the head and neck, and NPLs were mostly located in the lower extremity (p<0.001). The most common NPLs were benign nevus (18%, n=33) and pilomatrixoma (15%, n=27), while the most frequent IL was spongiotic/psoriasiform dermatitis (38%). Skin appendage/connective tissue tumors were the largest among NPLs (p=0.02). NPLs were more frequently seen in children >12 years old compared to ILs (p=0.03). The discordance rate between clinical and histopathological diagnoses was higher for NPLs (27% vs. 15%).

***Conclusion:*** Although the spectrum of skin lesions is broad in pediatric patients, most are benign in nature. The higher frequency of melanocytic and/or cystic lesions among children >12 years old may be attributed to increased self-care during puberty. Neoplastic/proliferative lesions of childhood seem to be less commonly recognized by clinicians, and a multidisciplinary approach remains the optimal method, considering the relatively high rate of discordance between the clinical and histopathological diagnoses.

## INTRODUCTION

The frequency of skin diseases in pediatric patients is affected by hereditary and environmental factors (1). The clinical presentation varies from localized subtle changes to generalized lesions affecting the patients’ quality of life (2). Some diagnoses require histopathological verification, and the correlation of clinical and histopathological findings is critical for diagnosis in certain diseases (3).

Both inflammatory and neoplastic skin disorders may occur in children. In fact, pediatric skin diseases have been reported to increase (4). However, there is limited data available on the epidemiological and clinicopathological evaluation of pediatric skin diseases.

In this study, we aimed to assess the clinicopathological features of neoplastic and non-neoplastic skin diseases in pediatric patients.

## MATERIAL and METHODS

### Case Selection

The study protocol has been approved by the institutional ethics committee (Approval date/no: 08.09.2021/192).

A total of 368 consecutive biopsies of 359 pediatric patients (i.e., < 18 years of age) that were evaluated between June 2020 and August 2021 in the Department of Pathology were included in the study. Clinicopathological data were retrospectively evaluated. Data on the age, sex, type and site of biopsy, size of the lesion, and preliminary clinical diagnosis were retrieved from hospital records, while histopathological diagnoses were retrieved from pathology reports. Patients with inadequate biopsy samples and/or samples with nonspecific findings were excluded.

### Statistical Analysis

Statistical analysis was performed using IBM SPSS Statistics for Windows, Version 22.0 (Chicago, IL). Descriptive statistical methods (mean, median, standard deviation, frequency, percentage, maximum-minimum) were used to evaluate the data. The Pearson chi-square test, the Fisher exact test, and the Freeman-Halton tests were used to analyze the relationship between clinical and pathological parameters. The histopathological diagnoses were evaluated and separated into two groups: neoplastic/proliferative lesions (NPLs) and non-neoplastic/inflammatory lesions (ILs). For statistical analyses, NPLs were grouped per their origin as follows: 1) tumors of the surface epithelium (verruca vulgaris, epidermal nevi, etc.), 2) melanocytic lesions, 3) tumors of the skin appendage/connective tissue (pilomatrixoma, dermatofibroma, etc.), 4) cutaneous cysts (epidermoid/trichilemmal cysts), 5) vascular lesions (lobular capillary, capillary, cavernous hemangioma, etc.), and 6) lymphoproliferative/related disorders. ILs were grouped based on their pattern as spongiotic/psoriasiform dermatitis, interphase dermatitis, superficial/deep perivascular dermatitis, vasculopathic reaction pattern, connective tissue diseases, dermal granulomatous/necrobiotic reaction pattern, and panniculitis. Patients were divided into three groups based on their age: 0-6 years, 7-12 years, and >12 years old. The preliminary clinical diagnoses and the final histopathological diagnoses were compared as “compatible” or “incompatible” in all biopsies. p <0.05 was considered as statistically significant.

## RESULTS

A total of 359 patients with single or multiple biopsy samples were evaluated. Nine patients had multiple biopsies from different sites with different diagnoses. We therefore noted them separately and there were 368 biopsies in total. 186 biopsies were in ILs, while 182 biopsies were in the NPLs group.

The mean age was 10.4±4.9 years (range 0-17 years). 51% of the cases (182 patients) were male, while 49% (177 patients) were female. The most common location was the lower extremity (31%, 114 patients). The other sites were the head and neck (26%), trunk (24%), and upper extremity (19%). Half of the biopsies were incisional (skin punch biopsy), while the other half was composed of excision materials. The number of patients with ILs was slightly higher than the cases with NPLs (51% (n=185) and 49% (n=174), respectively). There was no statistically significant difference between gender and NPLs/ILs distribution (p>0.05). NPLs were significantly more common in patients older than 12 years old (p=0.03) (Table 1).

**Table 1:** Demographic features of the patients for NPLs and ILs

		**NPLs (n= 174 patients)**	**ILs (n= 185 patients)**	**p value**
**Sex**	Male	55% (n=95)	47% (n=87)	
	Female	45% (n=79)	53% (n=98)	p>0.05
**Age groups**	0–6 years	21% (n=36)	30% (n=55)	
	7–12 years	33% (n=57)	32% (n=60)	
	>12 years old	46% (n=81)	38% (n=70)	p=0.03*

*Pearson chi-square test, p<0.05

**Figure 1:**
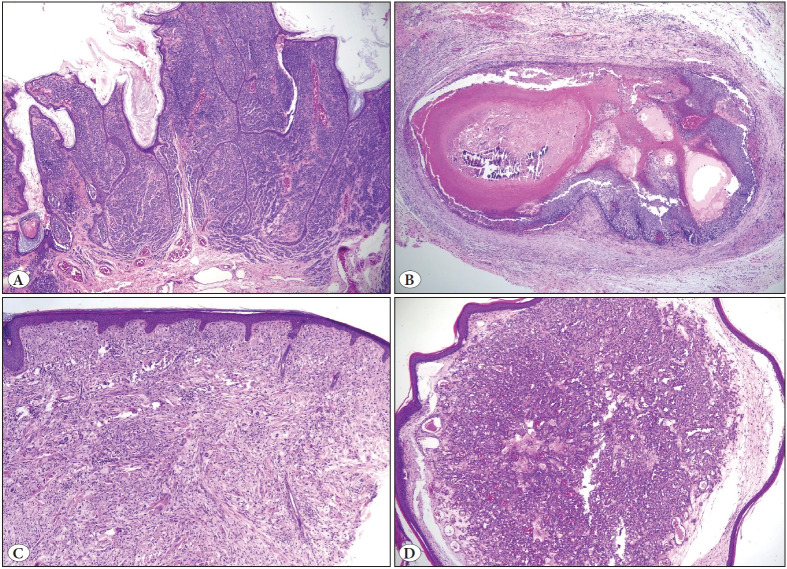
The most common NPLs in our series**. A)** Benign nevus, as the most common NPL; papillomatous compound nevus on the leg in a 17-year-old girl (40x). **B)** Pilomatrixoma, as the second most common NPL; composed of basaloid and ghost cells on the neck in a 6-year-old girl (40x). **C) J**uvenile xanthogranuloma on the neck of a 2-year-old child. Dermal infiltrate of foamy histiocytes with Touton type giant cells (40x), and **D)** Hemangioma, as the most clinically recognizable lesion in NPLs. Polypoid lobular capillary hemangioma on the neck of a 6-year-old girl (40x).

**Figure 2:**
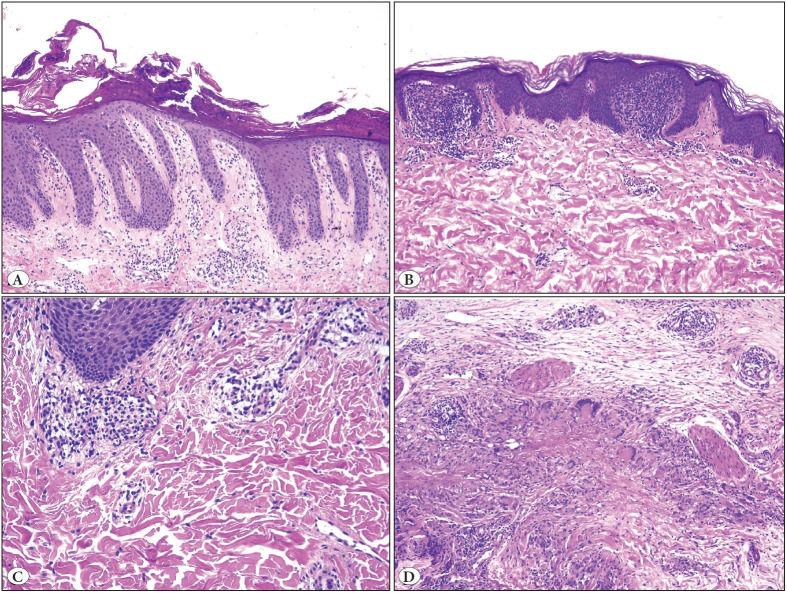
Inflammatory lesions (ILs). **A)** Spongiotic/psoriasiform dermatitis, as the most common IL; psoriasis vulgaris in a 16-year-old boy (100x). **B)** Lichen nitidus, as an interphase dermatitis, in a 5-year-old boy shows a ball and claw appearance at the dermoepidermal junction (100x). **C)** Perivascular lymphohistiocytic infiltrate in a 7-year-old girl (100x). **D)** Granuloma annulare in a 4-year-old girl, with degenerated collagen surrounded by lymphocytes, histiocytes, and multinucleate giant cells (100x).

NPLs were significantly more common in the head and neck, while the lower extremity was the most common site for ILs (p<0.001). The mean diameter of NPLs was 2.3 cm, the largest ones being connective tissue/skin appendage tumors (p=0.02). The most frequent NPLs were benign nevus (18%, n=33) and pilomatrixoma (15%, n=27) (Figure 1). Juvenile xanthogranuloma occurred only in children <6 years old (Table 2). The most common pattern of ILs was spongiotic/psoriasiform dermatitis (38%, n=71), followed by superficial/deep perivascular dermatitis (29%, n=55) (Figure 2, Table 3).

**Table 2:** The most common NPL diagnoses and distribution according to age groups.

**NPL groups and most common lesions (n=182)**	**0-6 years**	**7-12 years**	**>12 years**
**Tumors of the skin appendage/connective tissue (29%, n=54)**			
Pilomatrixoma (15%, n=27)	4	8	15
Sebaceous nevus (6%, n=11)	3	3	5
Other (8%, n=16)	4	7	5
**Vascular lesions (22%, n=40)**			
Lobular capillary hemangioma (12%, n=22)	1	10	11
Capillary hemangioma (6%, n=11)	6	3	2
Other (4%, n=7)	1	2	4
**Tumors of the surface epithelium (20%, n=36)**			
Verruca vulgaris (11%, n=20)	4	5	11
Epidermal nevus (3%, n=5)	0	2	3
Other (6%, n=11)	5	3	3
**Melanocytic lesions (18%, n=33)**			
Benign nevus (18%, n=33)	3	8	22
**Cutaneous cysts (8%, n=14)**			
Epidermal cyst (3%, n=5)	0	2	3
Trichilemmal cyst (2%, n=4)	0	1	3
Other (3%, n=5)	2	2	1
**Lymphoproliferative and related disorders (3%, n=5)**			
Juvenile xanthogranuloma (2.5%, n=4)	4	0	0
Mycosis fungoides (0.5%, n=1)	1	0	0

**Table 3:** The most common IL diagnoses and distribution according to age groups.

**IL groups and most common lesions (n=186)**	**0-6 years**	**7-12 years**	**>12 years**
**Spongiotic/psoriasiform dermatitis (38%, n=71)**			
Eczema (15%, n=28)	6	12	10
Psoriasis vulgaris (10%, n=19)	3	7	9
Other (13%, n=24)	7	10	7
**Superficial/deep perivascular dermatitis (30%, n=55)**			
Urticaria (10%, n=18)	6	2	10
Other (20%, n=37)	8	20	9
**Interphase dermatitis (12%, n=23)**			
Pityriasis lichenoides (4%, n=8)	1	3	4
Other (8%, n=15)	6	6	3
**Vasculopathic reaction pattern (9%, n=16)**			
Leukocytoclastic vasculitis (2%, n=3)	0	1	2
Other (7%, n=13)	0	5	8
**Connective tissue diseases (5%, n=10)**			
Morphea (2.5%, n=5)	0	1	4
Lichen sclerosus (2.5%, n=5)	0	3	2
**Dermal granulomatous/necrobiotic reaction patterns (4%, n=7)**			
Granuloma annulare (2%, n=3)	2	1	0
Other (2%, n=4)	0	2	2
**Panniculitis (2%, n=4)**			
Erythema nodosum (1%, n=2)	0	1	1
Other (1%, n=2)	0	1	1

The concordance of the clinical and pathological diagnoses was 73% in NPLs and 85% in ILs. The rate of clinicopathological discordance in NPLs was significantly higher than in ILs (p=0.03).

## DISCUSSION

Our study demonstrated that most pediatric skin lesions are benign in nature, and the most common NPLs are melanocytic nevus and pilomatrixoma. A broad spectrum of skin lesions may be encountered in pediatric patients, and this spectrum is affected by the geographic region (Table 4) (5–12), making a high frequency of melanocytic nevi unsurprising (7,9). Exposure to UV radiation and changes in a pre-existing nevus is the primary concern for the parents (1). Therefore, clinicians prefer to rule out the possible development of melanoma. Another reason for the relatively common excision of melanocytic nevus is cosmetic concerns. In fact, cosmetic disturbance is the main reason for the doctor’s visit for many skin lesions. Acquired skin disorders in puberty have a robust impact on self-esteem compared to congenital skin disorders (13,14). The higher frequency of melanocytic and/or cystic lesions among children >12 years old may also be attributed to increased self-care during puberty.

**Table 4:** The clinical and pathological data of the pediatric skin lesions from prior studies of different regions.

**Studies/country**	**Years**	**N**	**Age group**	**The most common skin lesions**	**The rate of the lesions**
Oyedepo et al. (Nigeria) (5)	2017-2018	1300	10-19 years	Disorders of appendages	43.2%
				Infections	33.2%
				Dermatitis	8.7%
Miotto et al. (Brazil) (6)	2017	2330	≤ 18 years	Inflammatory dermatoses	31.2%
				Genodermatoses	14.2%
				Infectious diseases	12.6%
Theiler et al. (Switzerland) (7)	2015-2017	506	≤ 16 years	Benign tumor/hamartoma/cyst	79%
				Dermatitis	18%
				Malignant tumor	2%
Afsar (Turkey) (8)	2004-2006	6000	≤ 18 years	Allergic skin diseases	49.9%
				Infectious diseases	20.5%
				Disorders of skin appendages	10.2%
Katsarou et al. (Greece) (9)	2005-2007	4071	≤ 12 years	Dermatitis/eczema	34.7%
				Infections	19.3%
				Nevi	5.6%
Sardana et al. (India) (10)	1997-2003	30078	≤ 12 years	Infections and infestations	47.1%
				Eczemas	26.9%
				Miliaria	5.4%
Hon et al. (China) (11)	2000	331	≤ 18 years	Eczema	33%
				Nevi	20%
				Viral warts	6%
Shibeshi (Ethiopia) (12)	1995-1997	1000	≤ 12 years	Allergic skin diseases	55%
				Infections	33%
				Photodermatosis	8%

Curiously, certain skin lesions exclusively occur in early childhood. Juvenile xanthogranuloma, a non-Langerhans cell histiocytosis of the first years of life, is one of them (15). Juvenile xanthogranuloma was diagnosed in only children that are younger than six years old in our series, consistent with the literature (16). Although it is usually a self-limiting benign lesion, some patients may manifest with a systemic disease that may require chemotherapy, or it may be associated with hereditary syndromes such as neurofibromatosis, etc. (17,18). Of note, the main challenge in the differential diagnosis of juvenile xanthogranuloma is Langerhans cell histiocytosis. These two histiocytic disorders may be distinguished based on their immunohistochemical profiles.

The most common location for NPLs was the head and neck in the present study. Similarly, Yang et al. found that the majority of the neoplastic lesions in pediatric patients were located in the scalp area (19). The authors also observed that congenital malformations, like nevus sebaceous, tend to occur in the scalp. Still, they might undergo a growth phase during late childhood due to the enlargement of sebaceous lobules. In our study, all sebaceous lesions were located on the scalp, but we did not observe any significant difference between age groups.

The frequency of inflammatory skin diseases was slightly higher (51%) in our study group, and spongiotic/psoriasiform dermatitis was the most common group, frequently including atopic/allergic dermatitis and psoriasis. This frequency seems to be higher than some of the previously reported data. Theiler et al. from Switzerland reported the rate of neoplastic lesions (benign tumor/hamartoma/cyst) as 79% and dermatitis as 18% in pediatric patients (7). In Katsarou et al.’s study from Greece, the rate of dermatitis/eczema was 34.7%, and rate of nevi was 5.6% in children (9). On the other hand, Afsar have found the rate of allergic skin diseases in childhood as 49.9% in their study from Turkey (8). In another study from Turkey, eczema was found at a rate of 14%, which was lower than in the studies from the same geographical region (20). Those differences may be attributable to populational immunologic differences, i.e., individuals from certain areas of the world may be more prone to allergic skin reactions and/or dermatitis. Eczema is the leading health issue for children in developed countries, while infections constitute a significant problem in developing countries (10,21). Moreover, in developed countries, multidisciplinary management and clinical follow-up of neoplastic skin lesions have become more critical nowadays (22). We think that being a large research hospital in a multicultural setting of a metropolis has also resulted in a similar frequency of NPLs and ILs in our study, as we receive excisional and incisional biopsies both from the Dermatology and Plastic Surgery Departments.

We observed that recognition of the NPLs seems to be more difficult for clinicians compared to ILs. This suggests that NPLs of childhood may have overlapping features clinically. The rates of clinicopathological compatibility were consistent with the literature both for NPLs and ILs (23,24). In prior studies about neoplastic skin disorders, the concordance rates were between 44% and 96.5% (25). On the other hand, Haugstved et al. found the rate of clinicopathological accuracy in non-neoplastic lesions as 57.5% (26). Although the concordance rates seem to differ widely among different clinical settings, they may be improved by a multidisciplinary approach and tight cooperation between the clinicians and the pathologists.

In conclusion, our study provides insight into both neoplastic and non-neoplastic skin lesions in children by histopathological confirmation. Although clinical and histopathological concordance is relatively high for specific types of lesions such as hemangiomas, a multidisciplinary approach is of utmost importance for the optimal management of skin lesions in children.

## Conflict of Interest

The authors declare that there is no conflict of interest.

## Funding

This research did not receive any specific grant from funding agencies in the public, commercial, or not-for-profit sectors.
